# Repetitive sequences and structural chromosome alterations promote intraspecific variations in *Zea mays* L. karyotype

**DOI:** 10.1038/s41598-020-65779-3

**Published:** 2020-06-01

**Authors:** Jéssica Coutinho Silva, Fernanda Aparecida Ferrari Soares, Mariana Cansian Sattler, Wellington Ronildo Clarindo

**Affiliations:** Laboratório de Citogenética e Citometria, Departamento de Biologia Geral, Centro de Ciências Biológicas e da Saúde, Universidade Federal de Viçosa, ZIP 36570-900 Viçosa, MG Brazil

**Keywords:** Cytogenetics, Plant genetics

## Abstract

LTR-retrotransposons, knobs and structural chromosome alterations contribute to shape the structure and organization of the *Zea mays* karyotype. Our initial nuclear DNA content data of *Z. mays* accessions revealed an intraspecific variation (2 C = 2.00 pg to 2 C = 6.10 pg), suggesting differences in their karyotypes. We aimed to compare the karyotypes of three *Z. mays* accessions in search of the differences and similarities among them. Karyotype divergences were demonstrated among the accessions, despite their common chromosome number (2n = 20) and ancestral origin. Cytogenomic analyses showed that repetitive sequences and structural chromosome alterations play a significant role in promoting intraspecific nuclear DNA content variation. In addition, heterozygous terminal deletion in chromosome 3 was pointed out as a cause of lower nuclear 2 C value. Besides this, translocation was also observed in the short arm of chromosome 1. Differently, higher 2 C value was associated with the more abundant distribution of LTR-retrotransposons from the family *Grande* in the karyotype. Moreover, heteromorphism involving the number and position of the 180-bp knob sequence was found among the accessions. Taken together, we provide insights on the pivotal role played by repetitive sequences and structural chromosome alterations in shaping the karyotype of *Z. mays*.

## Introduction

The genus *Zea* is a group of annual and perennial grasses native to a region extending from Mexico to Central America^1^. Based on morphological traits, geographical distribution and genomic data, five *Zea* species are currently recognized: the closely related perennial species *Zea diploperennis* Iltis, Doebley & Guzman (2n = 2×= 20) and *Zea perennis* (Hitchcock) Reeves & Mangelsdorf (2n = 4×= 40); the annual species *Zea luxurians* (Durieu & Ascherson) Bird; *Zea nicaraguensis* Iltis & Benz; and *Zea mays* L. (2n = 2×= 20). *Z. mays* is divided into four subspecies: (i) ssp. *parviglumis* Iltis & Doebley, which represents the direct progenitor of *Z. mays* ssp. *mays*^1–3^; (ii) ssp. *mays*, a well-studied cereal crop with extensive genetic diversity, commonly known as maize or corn^4^; (iii) ssp. *huehuetenangensis* (Iltis & Doebley) Doebley; and (iv) ssp. *mexicana* (Schrader) Iltis. Although controversies still exist regarding the origin of maize, allozyme genetic analyses^5,6^ and simple sequence repeat (SSR) markers^7^ have provided strong evidence to support *Z. mays* ssp. *parviglumis* as the progenitor of *Z. mays* ssp. *mays*.

Transposable elements (TEs) contribute to the dynamics of the nuclear genome, either through polymorphic insertions and deletions or by mediating ectopic recombination events that can drive structural variation in the genome^8^. TEs constitute over 85% of the maize reference (B73) genome^4^; of these, ~70% belong to Class-I long terminal repeat (LTR) retrotransposons, which replicate through an RNA intermediate, as in the superfamilies *Gypsy* and *Copia*^8^. The families *Huck, Cinful, Tekay/Prem-1* and *Grande* belong to the superfamily *Gypsy*, while *Prem-2/Ji* and *Opie* are included in *Copia*^8,9^. These families constitute a large fraction of the *Z. mays* genome and are distributed throughout its ten chromosomes^10^, but predominantly mapped in heterochromatic regions^9^. The cytogenetic determination of the genomic distribution of retrotransposons achieved to date in *Z. mays*^9,10^ has evinced their abundance and physical location in the chromosomes.

The genomic dynamism of the LTR-retrotransposon families, which has been characterized by amplification and loss, is responsible for the variation in genome size among *Z. mays* accessions^11,12^ that ranges from 2 C = 4.50 to 7.11 pg^13,14^. DNA content divergences have also been shown at chromosome level employing image cytometry. The DNA content has been measured for the long and short arms of each chromosome and the satellite of chromosome 6 of *Z. mays* ‘AL Bandeirante’^15^. The DNA content of chromosome 9 (2 C = 0.56 pg) was higher than that of chromosome 8 (2 C = 0.53 pg)^15^, a fact that can be related to accumulation of repetitive sequences in the knobs^16^. Hence, intraspecific variation in nuclear or chromosomal DNA content hints at karyotype differences, emphasizing the need to understand the causes of these variations in distinct *Z. mays* accessions.

In addition to TEs, knobs are also responsible for the intraspecific variation in *Z. mays* nuclear genome size^17,18^. Knobs are heterochromatic regions identified in pachytene and mitotic prometaphase and metaphase chromosomes by means of differential staining techniques^19^. They are composed of two tandem repeat sequences, of 180 base pairs (bp)^20^ and 350 bp (TR-1), besides harboring several LTR–retrotransposons^17,21^. The positions, number and size of the knobs are variable among both the accessions and the chromosomes of the same karyotype^9,22^. In some cases, the knobs might serve as chromosomal markers that provide physical evidence of crossing-over events between non-homologous chromosomes^23^. Despite the recognized role of TEs and knobs on the dynamism of *Z. mays* genome size variation, little is known about the important of these sequences on plant fitness. However, Bilinski *et al*.^18^ have found evidences that this variation may indeed be adaptive and that heterochromatic knob sequences are likely under the effect of natural selection. Therefore, considering that knob heteromorphism correlates with nuclear genome size, it is fundamental to map these portions in *Z. mays* chromosomes in order to verify their involvement in DNA content divergence.

In addition to LTR–retrotransposons and knobs, the chromosome structure and morphology can also be altered by structural rearrangements: duplication, deletion, translocations and/or inversions^24^. These rearrangements have been revealed in the genera *Solanum*^25^*, Brachypodium*^26^ and *Z. mays*^27^ by comparative cytogenetics via chromosome painting, thus assisting the elucidation of their evolutionary histories. During the process of double-strand break repair, several rearrangements may occur as a result of illegitimate recombination or through recombination of homologous ectopic sequences^28^. Thus, repetitive sequences such as TEs can provide a template for repairing the double-strand breaks. For this reason, heterochromatic regions rich in similar repetitive sequences are considered hotspots for double-strand breaks^29,30^.

Beyond the karyotype diversity and dynamism of *Z. mays* ssp. *mays*, this taxon also occupies a wide range of habitats and presents a diversity of morphological traits^1,31^, being a crop with several agricultural varieties specific for different uses^31^. For example, the popcorn is characterized by small, hard kernels that explode when heated, forming large flakes (*popping expansion*), the major feature that separates popcorn from other types of maize^31,32^. Sturtevant^33^ considered popcorn as a distinct species, *Zea everta*, which was posteriorly reduced to a subspecies^34^ and then considered as a mutant of flint maize^35^. However, archeological evidence and the quantitative trait of popping ability rendered improbable the hypothesis of a mutant origin from flint maize^32^. Currently, taxonomists consider that popcorn belongs to the taxon *Z. mays* ssp. *mays* (https://www.itis.gov/about_itis.html; http://www.plantsoftheworldonline.org). The origin and evolutionary relationship of popcorn with other types of maize remains unknown^31^. Therefore, the genomic *in situ* hybridization (GISH) and the comparative chromosome painting via chromosome-specific probes might to discriminate the homologous chromosome regions, contributing to understand the evolutionary relationships of popcorn.

Considering the remarkable karyotype dynamism, intraspecific variation in nuclear genome size and chromosomal DNA^15,36^ within *Z. mays*, the aim of this study was to perform a comparative analysis of the karyotypes of different *Zea* accessions, seeking to identify if the nuclear genome size variation among them is promoted by differential amounts of repetitive sequences and/or by structural chromosomal rearrangements.

## Results

G_0_/G_1_ peaks of *Z. diploperennis* and ‘Milho Pipoca Americano RS 20’ overlapped with the internal standard *Z. mays* ‘CE-777’, even with coefficients of variation between 2.91% and 4.77%. Because these overlapped G_0_/G_1_ peaks compromise the reliability, we also utilized the internal standard *S. lycopersicum*, thus avoiding this flow cytometry problem. *Z. diploperennis* showed 2 C = 5.76 ± 0.06 pg, corresponding to 2 C = 5.63 × 10^9^ bp, whereas ‘Milho Pipoca Americano RS 20’ showed mean 2 C = 5.55 ± 0.6 pg, corresponding to 2 C = 5.43 × 10^9^ bp. For the popcorn ‘15-1149-1’ and ‘AL Bandeirante’, the nuclear 2 C value was measured with the internal standard ‘CE-777’ and with *S. lycopersicum*. Popcorn ‘15-1149-1’ presented 2 C = 2.00 ± 0.17 pg, corresponding to 2 C = 1.96 × 10^9^ bp, and ‘AL Bandeirante’ showed 2 C = 6.10 pg, corresponding to 2 C = 5.97 × 10^9^ bp (Supplementary Fig. 1). Thus, the mean 2 C value of *Z. diploperennis* was 2 C = 3.76 pg higher than ‘15-1149-1’, 2 C = 0.21 pg higher than ‘Milho Pipoca Americano RS 20’, and 2 C = 0.34 pg lower than ‘AL Bandeirante’. ‘Milho Pipoca Americano RS 20’ presented 2 C = 3.55 pg higher than ‘15-1149-1’, and 2 C = 0.55 pg lower than ‘AL Bandeirante’. In a similar trend, ‘15-1149-1’ exhibited 2 C = 4.10 pg lower than ‘AL Bandeirante’.

Given these nuclear genome size differences, we explored the karyotypes in order to understand the causes of these divergences among *Zea* accessions. For this, a metaphasic index of 60% was obtained from the cell cycle arrest, achieved by treatment involving hydroxyurea followed by amiprophos-methyl. Besides, to ensure morphologically preserved chromosomes with well-defined telomeres and primary constrictions, enzymatic maceration of root meristems and air-drying technique were used for slide preparation.

**Figure 1:**
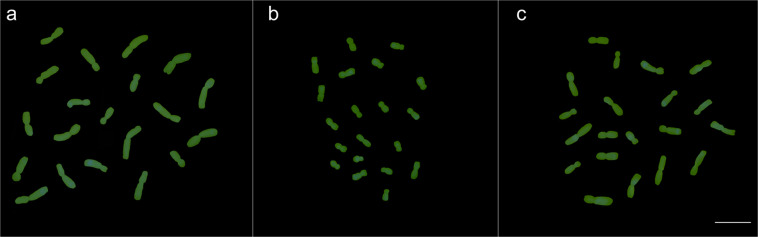
GISH in metaphase chromosomes of *Z. mays* labelled with ChromaTide-488-5-dUTP (green). **(a)** ‘AL Bandeirante’ chromosomes fully labelled by the genomic probe from ‘Milho Pipoca Americano RS 20’. **(b) ‘**AL Bandeirante’ and **(c)** ‘Milho Pipoca Americano RS 20’ chromosomes fully labeled by the genomic probe of *Z. diploperennis*. Bar = 10 µm. Images were digitized using the Image-Pro Plus software version 6.1 (https://www.mediacy.com/imageproplus).

Once the differences in nuclear genome size were verified, the genomic homology among *Z. mays* accessions was confirmed. Even with GISH stringency at 85–90%, all chromosomes of at least ten ‘Milho Pipoca Americano RS 20’ and ‘AL Bandeirante’ metaphases were fully hybridized by the genomic probe of *Z. diploperennis*. The same result was observed for the genomic probe of ‘Milho Pipoca Americano RS 20’ applied to the ‘AL Bandeirante’ karyotype (Fig. 1). From hybridization of our previously constructed probe of *Z. mays* ‘AL Bandeirante’ chromosome 1, the homology between *Z. mays* accessions was also evidenced by specific painting of the chromosome 1 of ‘Milho Pipoca Americano RS 20’ (Supplementary Fig. 2). Therefore, these results provide substantial evidence that popcorn belongs to the subspecies *mays*.

**Figure 2:**
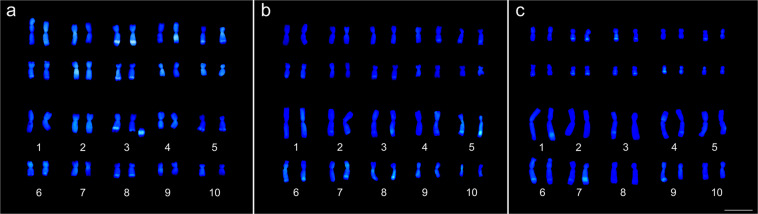
Differential DAPI staining (blue) in two metaphases of **(a)** ‘15-1149-1’, **(b)** ‘Milho Pipoca Americano RS 20’ and **(c)** ‘AL Bandeirante’. **(a)** Structural chromosome alterations in ‘15-1149-1’: translocation in the short arm of chromosome 1 and heterozygous terminal deletion in the long arm of chromosome 3. **(b,c)** In ‘Milho Pipoca Americano RS 20’ and ‘AL Bandeirante’, no structural chromosomal aberration was observed. The DAPI-banding pattern in *Z. mays* chromosomes was promoted by the preferential binding of this fluorochrome to A-T rich sequences, allowing to evidence the knob portions in a cyan blue color. Bar = 10 µm. Images were digitized using the Image-Pro Plus software version 6.1 (https://www.mediacy.com/imageproplus).

Nevertheless, karyotype differences were demonstrated among the *Z. mays* ssp. *mays* accessions. Structural chromosome changes were identified in all karyotypes of ‘15-1149-1’, which were specifically stained by Feulgen reaction, DAPI and labeled by the 180-bp probe. Two alterations were identified (Supplementary Fig. 3): terminal deletion in the long arm of chromosome 3 and translocation in the short arm of chromosome 1 (Fig. 2a). The translocation was identified as one detectable chromosome fragment, but it was not classified as non-reciprocal or reciprocal. Differently, no structural chromosomal aberration was observed in ‘Milho Pipoca Americano RS 20’ and ‘AL Bandeirante’ karyotypes (Fig. 2b,c).

**Figure 3:**
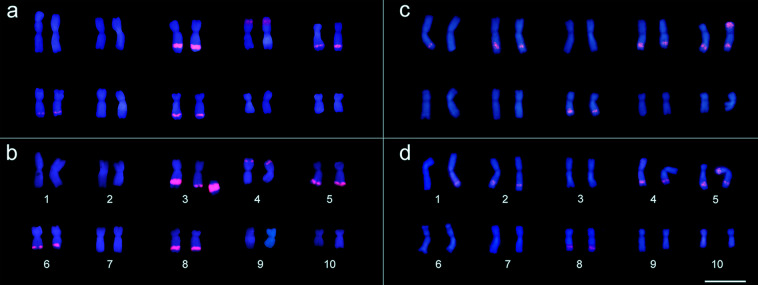
180-bp sequence site mapping from probe labelled with Tetramethylrhodamine 5-dUTP (red) in metaphases of **(a,b)** ‘15-1149-1’ and **(c,d)** ‘AL Bandeirante’. **(a,b)** In ‘15-1149-1’, 180-bp was mapped in the interstitial portion of the long arm in chromosomes 3, 5, 6 and 8, and in the terminal portion of the short arm on chromosome 4. **(a)** Note the translocation in the short arm of the chromosome 1, and **(b)** the heterozygous terminal deletion in the chromosome 3 long arm around the large block of 180-pb sequence. **(c,d)** In ‘AL Bandeirante’, 180-bp sequence was mapped on in the interstitial portion of the long arm in chromosomes 1, 2, 4, 5 and 8, and in the terminal portion of the short arm in chromosome 5. Note the heterozygosity of the 180-bp sequence in the chromosomes 1 and 5. Bar = 10 µm. Images were digitized using the Image-Pro Plus software version 6.1 (https://www.mediacy.com/imageproplus).

**Figure 4:**
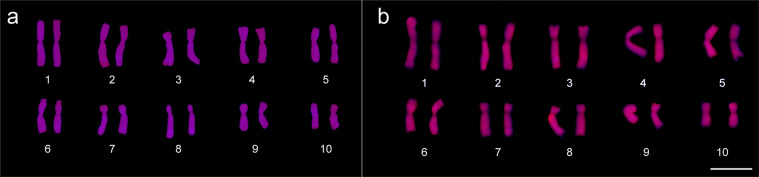
Mapping of the *Grande* LTR-retrotransposon sequence, from probes labelled with Tetramethylrhodamine 5-dUTP (red), in chromosomes of **(a)** ‘Milho Pipoca Americano RS 20’ and **(b)** ‘AL Bandeirante’. **(a)** Hybridization signals were fully labeled in the ten chromosomes, telomere to telomere, in all metaphases of ‘Milho Pipoca Americano RS 20’. **(b)** In ‘AL Bandeirante’ metaphases, the chromosomes displayed strong hybridization signals. Bar = 10 µm Images were digitized using the Image-Pro Plus software version 6.1 (https://www.mediacy.com/imageproplus).

Apart from these structural chromosome aberrations, karyotype variations were also found regarding the number and position of the 180-bp knob sequence, including heteromorphism within the same karyotype between the chromosome pair (Fig. 3). The 180-bp sequence was mapped in nine different chromosome portions (1 L, 2 L, 3 L, 4 S, 4 L, 5 S, 5 L, 6 L or 8 L) in the karyotypes of *Z. mays* ‘15-1149-1’ and ‘AL Bandeirante’. *Z. mays* ‘15-1149-1’ exhibited positive signals in the chromosome portions 3 L, 4 S, 5 L, 6 L and 8 L (Fig. 3a,b), whereas ‘AL Bandeirante’ displayed signals in 1 L, 2 L, 4 L, 5 S, 5 L and 8 L, also presenting heterozygosity for the presence/absence of signals in the chromosome pairs 1 and 5 (Fig. 3c,d). The karyotypes of the analyzed *Z. mays* accessions also differed in relation to *Grande* LTR–retrotransposon mapping. Uniform hybridization signals from this probe were obtained in ten metaphases of ‘Milho Pipoca Americano RS 20’ (Fig. 4a). The ‘AL Bandeirante’ karyotype exhibited stronger hybridization signals throughout the chromosome length in 15 metaphases (Fig. 4b), indicating that ‘AL Bandeirante’ possesses more copies of this LTR–retrotransposon.

## Discussion

Comparing the nuclear DNA content of *Z. diploperennis* (2 C = 5.76 pg), ‘Milho Pipoca Americano RS 20’ (2 C = 5.55), ‘15-1149-1’ (2 C = 2.00 pg) and ‘AL Bandeirante’ (2 C = 6.10 pg), a difference of up to 4.10 pg was found. Considering the chromosomal DNA content of ‘AL Bandeirante’, and according to mean values reported by Silva *et al*.^15^, the 4.10 pg corresponds to approximately five times the chromosome 1 (2 C = 0.80 pg = 4.00 pg) or ten times the chromosome 10 (2 C = 0.38 pg = 3.80 pg). These data reinforce the intraspecific variation in nuclear DNA content observed in *Z. mays*, which has been reported to range from 2 C = 4.50 pg to 7.11 pg^13,14^. Given this variation, karyotype differences and similarities were sought inside each *Z. mays* accession studied here.

Based on divergences regarding nuclear genome size, the first step was to verify the evolutionary relationship of the *Z. mays* spp. *mays* accessions. Genomic probes of *Z. diploperennis* provided hybridization signals, from telomere to telomere, in the chromosomes of ‘AL Bandeirante’ and ‘Milho Pipoca Americano RS 20’, confirming the genomic affinity of these accessions to the basal species *Z. diploperennis*^1^. Hybridization signals were also observed over all *Z. mays* ssp. *mays* chromosomes with the genomic probe of *Z. diploperennis*, but they were weaker than those detected with genomic probes of *Z. mays* spp. *mexicana* and *Z. mays* spp. *parviglumis*^37^, which are more phylogenetically close to *Z. mays* spp. *mays*^1^. Reinforcing the evolutionary relationship, the F_1_ hybrids of *Z. diploperennis* x *Z. mays* ssp. *mays* presented regular meiotic chromosome pairing and high pollen viability^38,39^.

The genome homology between ‘AL Bandeirante’ and ‘Milho Pipoca Americano RS 20’ was also endorsed by chromosome painting of the chromosome 1. Using the application proposed by Soares *et al*.^40^, chromosome painting between ‘AL Bandeirante’ and ‘Milho Pipoca Americano RS 20’ was informative in comparative karyotype analysis, reflecting the common evolutionary origin of these accessions. This approach has been successfully used for the resolution of phylogenetic questions in *Brachypodium* genus^26^. The GISH and chromosome painting data obtained here confirmed that popcorn belongs to *Z. mays* ssp. *mays*.

After confirming the evolutionary origin, some karyotype divergences were evidenced among the *Z. mays* accessions, as well as differences between homologue chromosome pairs in the same karyotype, providing cytogenetic data to understand the intraspecific variation in nuclear DNA content. Translocation and terminal deletion were distinguished in ‘15-1149-1’, which resulted in a morphological change in the chromosomes 1 and 3, respectively. Thus, cytogenetic preparations applying cell dissociation combined with air-drying technique were considered essential for the correct interpretation of these karyotype changes, since this methodology replaces the squashing step that can promote chromosome breakages^41^. The terminal deletion in the long arm of chromosome 3 occurred around the knob, which is considered a hotspot of chromosome structure alterations. The knobs present a complex organization, in which blocks of 180-bp sequence are interrupted by LTR–retrotransposons^21^. Structural chromosomal rearrangements occur within regions composed of repetitive DNA sequences^24,30^. In addition, Lysák and Schubert^42^ related that repetitive sequences scattered throughout the genome, especially TEs, are involved in various chromosomal rearrangements, such as deletion and translocation, because ectopic homologous sequences provide a template for recombination during the repair of double-strand breaks, a phenomenon denominated *ectopic recombination*.

The translocation and terminal deletion found in ‘15-1149-1’ represent one of the causes associated to the relatively lower nuclear DNA content (2 C = 2.00 pg) of this accession in relation to the others. In *S. lycopersicum*, a difference of 2 C = 0.09 pg between the wild type and the mutant ‘BHG 160’ was found to be due to a heterozygous terminal deletion in the short arm of the chromosome 1^43^. The translocation is another karyotype aberration that includes a broken chromosome, resulting in a chromatid fragment. However, differently from a deletion or inversion, the chromatid fragment moves and joins the homologue pair or another chromosome^44^. Therefore, the translocation in the short arm of chromosome 1 also evidences that chromosome breakage occurred in the ‘15-1149-1’ karyotype.

These structural chromosome aberrations were highlighted in ‘15-1149-1’ and associated to low nuclear DNA content, yet other karyotype divergences were found from the mapping of the 180-bp sequence and *Grande* LTR-retrotransposon. Intraspecific variation in nuclear DNA content was also an outcome of the number and heterozygosity of the 180-bp sequence in the karyotype, as well as of the *Grande* LTR-retrotransposon signals found among the *Z. mays* accessions. This result shows that repetitive sequences typical of heterochromatin portions, LTR-retrotransposons and 180-bp knobs, are also responsible for genome size variation within *Z. mays*. The increase in nuclear genome size in this species has also been correlated with 180-bp knob abundance^45^. Furthermore, Bilinski *et al*.^18^ demonstrated that the variations in nuclear genome size are driven by natural selection, causing changes in the abundance of repeat sequences across the genome of *Z. mays*, as significant reductions in heterochromatic knobs. Knobs, which are constituted by 180-bp and 350-bp sequences, are polymorphic in relation to their number, size and distribution across the ten *Z. mays* chromosomes^17,46^, affecting 5–20% of the length of the chromosome arm^47^. Besides, the heterozygosity observed in the chromosome portions 1 L and 5 S only in ‘AL Bandeirante’ is also a karyotype evidence of the differential accumulation of the 180-bp sequence, and consequent change in the chromosomal DNA content among accessions. A heterozygous condition has been appointed as a cause of crossing-over suppression^48^. Although the origin of this polymorphism is still uncertain, it has been presumed that knobs can move around according to the “complex megatransposons” hypothesis^49^. This hypothesis proposes that TR-1 tandem repeat sequences are capable of forming fold-back DNA segments, driving the knobs in the *Z. mays* genome^49^.

Regarding the distribution of the *Grande* LTR-retrotransposon, which belongs to the *Gypsy* superfamily, ‘AL Bandeirante’ stood out with stronger hybridization signals than ‘Milho Pipoca Americano RS 20’. In addition to this mapping, represented by karyograms, the influence of the *Grande* LTR-retrotransposon sequence on nuclear DNA content among *Z. mays* accessions was also demonstrated. *Grande* LTR-retrotransposon distribution produced a uniform hybridization pattern along the extension of metaphasic *Z. mays* chromosomes, differently from was reported by Mroczek and Dawe^10^ and Lamb *et al*.^9^ that found a speckled hybridization pattern along the chromosome extension. Distribution of the *Gypsy* LTR-retrotransposon in different chromosome regions has also been reported for other species. In *Arabidopsis thaliana* (L.) Heynh^50^ and *Asparagus officinalis* L.^24^, this LTR is mainly distributed in the centromeres. Differently, in *Silene latifolia* Poir., the signals for this LTR were observed in subtelomeric heterochromatin regions of the chromosomes^51^.

Many mechanisms have shaped the karyotype organization in plants, such as LTR-retrotransposons^8^. The higher 2 C value and more *Grande* LTR-retrotransposon signals in ‘AL Bandeirante’ than in ‘Milho Pipoca Americano RS 20’ reflect the consequences of the LTR-retrotransposon dynamism – amplification and/or loss. Increase in nuclear genome size is promoted by a *de novo* DNA sequence of the retrotransposon that is inserted into the genome after an RNA intermediate to be converted into a cDNA molecule by the reverse transcriptase^4,52^. On the other hand, unequal and illegitimate recombination are associated with a high frequency of genomic DNA loss, and may counterbalance the amplification of LTR-retrotransposons^53^, as reported for *Arabidopsis*^54^ and *Oryza sativa* L.^55^.

## Conclusions

Cytogenomic analysis showed that the intraspecific nuclear genome size variation in *Z. mays* spp. *mays* accessions was promoted by structural chromosome alterations and repetitive sequences. Considering the genomic relationship between the accessions, the dynamic genome of *Z. mays* was newly demonstrated by occurrence of terminal deletions, translocations, 180-bp knob sequence and *Grande* LTR-retrotransposon. Therefore, multiple karyotype factors are related to the changes in *Z. mays* and should be explored in other plant species.

## Material and Methods

### Plant material

Seeds of *Z. diploperennis* and *Z. mays* ‘15-1149-1’ (popcorn) were provided by the Maize Germplasm Bank of the Embrapa Maize and Sorghum (Sete Lagoas, Minas Gerais – Brazil) and Dr. Marcelo Soriano Viana (Universidade Federal de Viçosa, Minas Gerais – Brazil), respectively. Commercial seeds of *Zea mays* ‘AL Bandeirante’ and ‘Milho Pipoca Americano RS 20’ were also used. Seeds of the flow cytometry standards *Solanum lycopersicum* L. ‘Stupické’ and *Zea mays* ‘CE-777’ were provided by Dr. Jaroslav Doležel (Experimental Institute of Botany – Czech Republic). According to the *Zea* phylogeny proposed by Hufford *et al*.^1^ based on data from ~1000 SNPs by Fang *et al*.^3^, *Z. diploperennis* is basal in relation to *Z. mays* spp. *mays*. Therefore, this species was used to compare the nuclear genome size and to construct genomic probes in order to verify the ancestral relationship and homology among *Z. mays* spp. *mays* accessions.

### Nuclear genome size

In order to avoid G_0_/G_1_ peak overlapping in flow cytometry histograms due to close nuclear DNA content, the nuclear genome sizes of *Z. diploperennis*, ‘AL Bandeirante’, ‘Milho Pipoca Americano RS 20’ and ‘15-1149-1’ (samples) were measured using the reference standards *S. lycopersicum* or *Z. mays* (2 C = 2.00 pg and 2 C = 5.55 pg, respectively; Praça-Fontes *et al*.^56^. Leaf fragments from each sample and each internal standard (*S. lycopersicum* or *Z. mays*) were co-chopped^57^, and the nuclei were isolated and stained using Otto buffers^58^, following the procedure proposed by Praça-Fontes *et al*.^56^. The nuclei suspensions were stained with propidium iodide and analyzed in a BD Accuri C6 flow cytometer (Accuri cytometers, Belgium) equipped with a laser source to detect emissions at FL3 (>670 nm). The histograms were analyzed using the BD CSampler software. Four technical replicates were performed for each sample with each standard, analyzing over 10,000 nuclei each time. The mean 2 C nuclear genome size was measured for each *Zea* sample by dividing the mean channel of the fluorescence peak corresponding to the standard’s G_0_/G_1_ nuclei by that of each sample.

Due to the intraspecific variation in mean 2 C value among the *Z. mays* accessions, the karyotypes were characterized with the aim of identifying possible differences and similarities among them. For this, the 180-bp knob sequence and *Grande* LTR–retrotransposon were mapped via fluorescence *in situ* hybridization (FISH). In addition, GISH using the genomic DNA of the wild related species *Z. diploperennis* was performed to confirm the evolutionary origin of popcorn. Owing to constraints in seed availability and low germination rate, ‘15-1149-1’ was replaced by ‘Milho Pipoca Americano RS 20’.

### Chromosome preparation

Roots of all accessions showing 1 cm in length were incubated for 18 h in 0.20 g L^−1^ MS salts (Sigma) and 1.75 mM hydroxyurea (inhibitor of ribonucleotide reductase, Sigma) at 30 °C. The roots were washed in dH_2_O for four times of 15 min, and then treated with 3 µM amiprophos-methyl (inhibitor of microtubule polymerization, Sigma) for 4 h at 30 °C. Later, the roots were fixed in 3:1 methanol: acetic acid solution, with three changes of 10 min each, and stored at −20 °C. Afterwards, the roots were again washed for three times in dH_2_O, then macerated for 2 h at 36 °C in enzymatic pool (4% cellulase Sigma, 0.4% hemicellulase Sigma, 1% macerozyme Onozuka R10 Yakult, 100% pectinase Sigma) diluted in dH_2_O in the proportion 1:8 (enzyme pool: dH_2_O). After the maceration procedure, the roots were washed in dH_2_O, fixed in 3:1 methanol: acetic acid solution and stored at −20 °C^15^. From the macerated root meristems, slides were prepared by cellular dissociation and air-drying techniques^41^. The slides were chosen for FISH and GISH based on their number of metaphases with morphologically preserved chromosomes, exhibiting well-defined telomere and primary constriction.

Slides showing karyotypes with possible structural chromosome alterations were subjected to Feulgen reaction, a method employed to stoichiometrically and specifically stain the DNA. Thereby, as done by Silva *et al*.^15^, selected slides were immediately placed in a fixative solution of methanol: 37% formaldehyde: acetic acid (17:5:1) for 24 h at 25 °C. After fixation, the slides were washed in dH_2_O, air-dried, hydrolyzed in 5 M HCl for 18 min at 25 °C, and stained with Schiff’s reagent (Merck) for 16 h at 4 °C.

### Genomic homology among *Z. mays* accessions

Genomic DNA of ‘Milho Pipoca Americano RS 20′ and *Z. diploperennis* were obtained according to Doyle and Doyle^59^. DNA concentration and purity were determined by spectrophotometry using NanoDrop (Invitrogen), and DNA integrity was further verified by 1.5% agarose gel electrophoresis. The genomic DNA was amplified and labeled by degenerate oligonucleotide-primed polymerase chain reaction (DOP-PCR). The amplification reaction mix consisted of 4 μM degenerated oligonucleotide primer (DOP 5′-CCGACTCGAGNNNNNNATGTGG-3′), 200 ng of genomic DNA of ‘Milho Pipoca Americano RS 20’ or *Z. diploperennis*, 200 μM of each dNTP (Promega), 1X polymerase buffer, and 2.5 U AccuTaq LA DNA Polymerase (Sigma). The labeling reaction consisted of 4 μM DOP, 200 ng of the genomic DNA, 200 μM each of dATP, dCTP and dGTP, 150 μM dTTP, 50 μM ChromaTide Alexa Fluor 488-5-dUTP (Life Technologies), 1X enzyme reaction buffer (Sigma), and 2.5 U AccuTaq LA DNA Polymerase (Sigma). The PCR conditions were: 96 °C for 3 min; 30 cycles of denaturation at 91 °C for 1 min; 56 °C for 1 min; increments of 0.1 °C/s until 68 °C; and 68 °C for 5 min. The labeled genomic probes were quantified in a NanoDrop spectrophotometer (Invitrogen) and evaluated by electrophoresis in 1.5% agarose gel. The amplified fragments ranged from 200 to 700 bp. The genomic DNA probe of *Z. diploperennis* was hybridized in ‘AL Bandeirante’ and ‘Milho Pipoca Americano RS 20’, and the genomic DNA probe of ‘Milho Pipoca Americano RS 20’ was hybridized in ‘AL Bandeirante’.

Recently, our research group constructed a chromosome-specific probe for the chromosome 1 of *Z. mays* ssp. *mays* ‘AL Bandeirante’^40^, which was used for chromosome painting in *Z. mays*. DNA from the chromosome 1, previously amplified by DOP-PCR as reported in Soares *et al*.^40^, was used in a new labeling reaction. The PCR program and labeling of the amplified fragment were performed as described above. The probe obtained from chromosome 1 was evaluated by electrophoresis in 1.5% agarose gel, showing fragments ranging from 100 to 900 bp.

### Mapping of the 180-bp knob sequence and Grande LTR*–*retrotransposon

The *Grande* LTR–retrotransposon probe was generated by PCR using the primers *F*: 5′-TGCGAGGATAAGTCGGCGAAG-3′ and *R*: 5′-GGTGTTTTTAGGAGTAGGACGGTG-3′^10^. This family was selected for its wide distribution in the *Z. mays* genome. The probe of the 180-bp knob sequence was amplified from the primers *F*: 5′-ATAGCCATGAACGACCATTT-3′ and *R*: 5′-ACCCCACATATGTTTCCTTG-3′^14^. The reaction mixture consisted of: 0.5 μM of each primer, 200 ng of genomic DNA, 200 μM of each dNTP (Promega), 1X reaction buffer (Invitrogen), 2 mM MgSO_4_ (Invitrogen), and 2 U Platinum Taq DNA Polymerase High Fidelity (Invitrogen). The PCR conditions for the *Grande* LTR–retrotransposon were as follows: initial denaturation at 95 °C for 5 min; 30 cycles of denaturation at 95 °C for 1 min; annealing at 66 °C for 1 min; extension at 68 °C for 1 min and 30 sec; and final extension at 68 °C for 5 min. For the 180-bp sequence, amplification conditions were the following: initial denaturation at 95 °C for 5 min; 30 cycles of denaturation at 95 °C for 1 min; annealing at 47 °C for 1 min; extension at 68 °C for 1 min and 30 sec; and final extension at 68 °C for 5 min. The labeling reaction consisted of 0.5 μM of each primer, 200 ng of the amplified DNA, 200 μM each of dATP, dCTP and dGTP, 150 μM dTTP, 40 μM Tetramethylrhodamine 5-dUTP (Roche), 1X of the enzyme reaction buffer (Invitrogen), and 2.5 U AccuTaq LA DNA Polymerase. The PCR conditions were the same as described above for each sequence.

### In situ hybridization

The procedures were performed as described by Soares *et al*.^40^ and Schwarzacher and Heslop-Harrison^60^, with modifications. Briefly, the slides were washed in 1X PBS buffer for 5 min, fixed with 4% formalin for 15 min, washed again in 1X PBS for 5 min, and dehydrated in cold ethanol series (70%, 85% and 100%) for 5 min each. Chromosome denaturation was carried out in 70% formamide/2X saline-sodium citrate (SSC) buffer for 3 min, at 68 °C for ‘Milho Pipoca Americano RS’ and ‘15-1149-1’ and 70 °C for ‘AL Bandeirante’. The difference in temperature is due to over-denaturation when popcorn chromosomes were submitted to 70 °C. Subsequently, the slides were dehydrated in cold ethanol series (70%, 85% and 100%). The hybridization mixture consisted of 50% formamide (Sigma) + 2X SSC (Sigma), 35 μg competitor DNA (Herring Sperm DNA, Promega) and 200 ng of the probe, with denaturation at 85 °C for 5 min followed by immediate transfer to ice. Slides were incubated with 35 μL hybridization mixture, covered by plastic coverslip HybriSlip (Sigma) and sealed with Rubber Cement (Elmer’s). The hybridization procedure was conducted in a ThermoBrite system (ThermoFisher) at 37 °C for 24 h. After this period, stringency washes were performed in three solutions of 50% formamide/2X SSC and one of 2X SSC, for 5 min each, at 42 °C for ‘Milho Pipoca Americano RS’ and ‘15-1149-1’ and 45 °C for ‘AL Bandeirante’. Metaphases were counterstained with 40% glycerol/PBS + 6-diamidino-2-phenylindole (DAPI). The same slides used for FISH mapping of the 180-bp knob sequence were also used to evaluate the differential DAPI banding pattern.

The images were captured with a digital video camera 12-bit CCD (Olympus) coupled to a photomicroscope Olympus BX-60 equipped with epifluorescence and immersion objective of 100×, numeric aperture of 1.4. The frame was digitized using the Image-Pro Plus 6.1 software (Media Cybernetics).

## Supplementary information

Supplementary information.
