# Author Correction: Repetitive sequences and structural chromosome alterations promote intraspecific variations in *Zea mays* L. karyotype

**DOI:** 10.1038/s41598-020-67919-1

**Published:** 2020-06-26

**Authors:** Jéssica Coutinho Silva, Fernanda Aparecida Ferrari Soares, Mariana Cansian Sattler, Wellington Ronildo Clarindo

**Affiliations:** Laboratório de Citogenética e Citometria, Departamento de Biologia Geral, Centro de Ciências Biológicas e da Saúde, Universidade Federal de Viçosa, Viçosa, MG ZIP 36570-900 Brazil

Correction to: *Scientific Reports* 10.1038/s41598-020-65779-3, published online 01 June 2020

This Article contains a typographical error in the Methods section under subheading ‘In situ hybridization’, whereby;

“Metaphases were counterstained with 40% glycerol/PBS + 6-diamidino-2-phenylindole (DAPI).”

should read:

“Metaphases were counterstained with 40% glycerol/PBS + 4′,6-diamidino-2-phenylindole (DAPI).”

