# Supplementary material for: Repetitive sequences and structural chromosome alterations promote intraspecific variations in Zea mays L. karyotype
Source: Sci Rep. 2020 Jun 1;10:8866. doi: 10.1038/s41598-020-65779-3 (PMC7264354; doi:10.1038/s41598-020-65779-3)
Supplement: Supplementary file 1 — Supplementary information. [file 41598_2020_65779_MOESM1_ESM.pdf]

Repetitive sequences and structural chromosome alterations promote intraspecific variations in *Zea mays* L. karyotype.

Authors: Jéssica Coutinho Silva[1*], Fernanda Aparecida Ferrari Soares[1], Mariana Cansian Sattler[1], Wellington Ronildo Clarindo[1]

[1]Laboratório de Citogenética e Citometria, Departamento de Biologia Geral, Centro de Ciências Biológicas e da Saúde, Universidade Federal de Viçosa. ZIP 36570-900 Viçosa – MG, Brazil.

*Corresponding author e-mail: coutinho.silva530@gmail.com

Tel: + 55 (31) 3612-5005; Fax: + 55 (31) 3612-5028

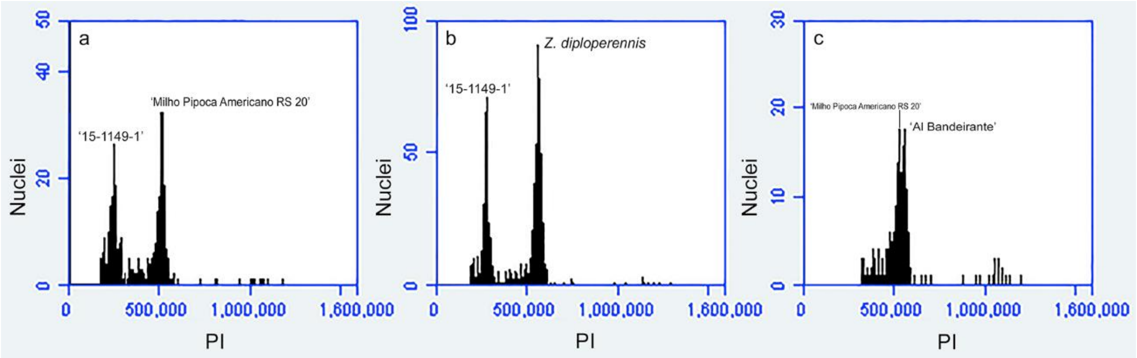

Supplementary Figure 1 – Nuclear DNA content of the *Z. mays* spp. *mays* accessions and *Z. diploperennis*. **(a)** '15-1149-1' G_0/G_1 nuclei peak 2C = 2.00 pg and 'Milho Pipoca Americano RS 20' G_0/G_1 nuclei peak 2C = 5.55 pg. **(b)** '15-1149-1' G_0/G_1 nuclei peak 2C = 2.00 pg and *Z. diploperennis* G_0/G_1 nuclei peak 2C = 5.76 pg. **(c)** 'Milho Pipoca Americano RS 20' G_0/G_1 nuclei peak 2C = 5.55 pg and 'AL Bandeirante' G_0/G_1 nuclei peak 2C = 6.10 pg. Histograms were generated using BD Csampler software version Accuri C6.

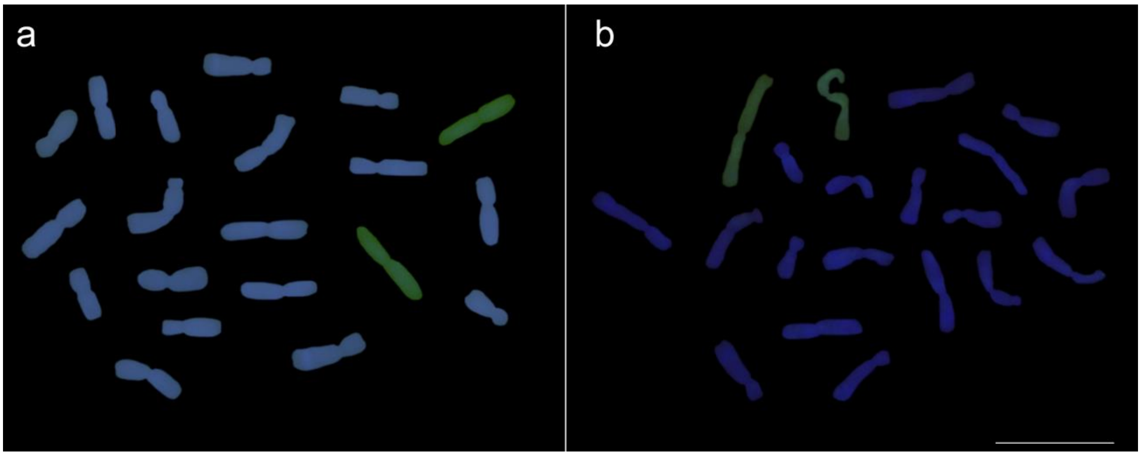

Supplementary Figure 2 – Chromosome painting in *Z. mays* **(a)** 'Milho Pipoca Americano RS 20' and **(b)** 'AL Bandeirante' using chromosome-specific probe constructed for Chromosome 1 of *Z. mays* labelled with ChromaTide-488-5-dUTP (green). Bar = 10 µm. Images were digitized using the Image-Pro Plus software version 6.1 (https://www.mediacy.com/imageproplus).

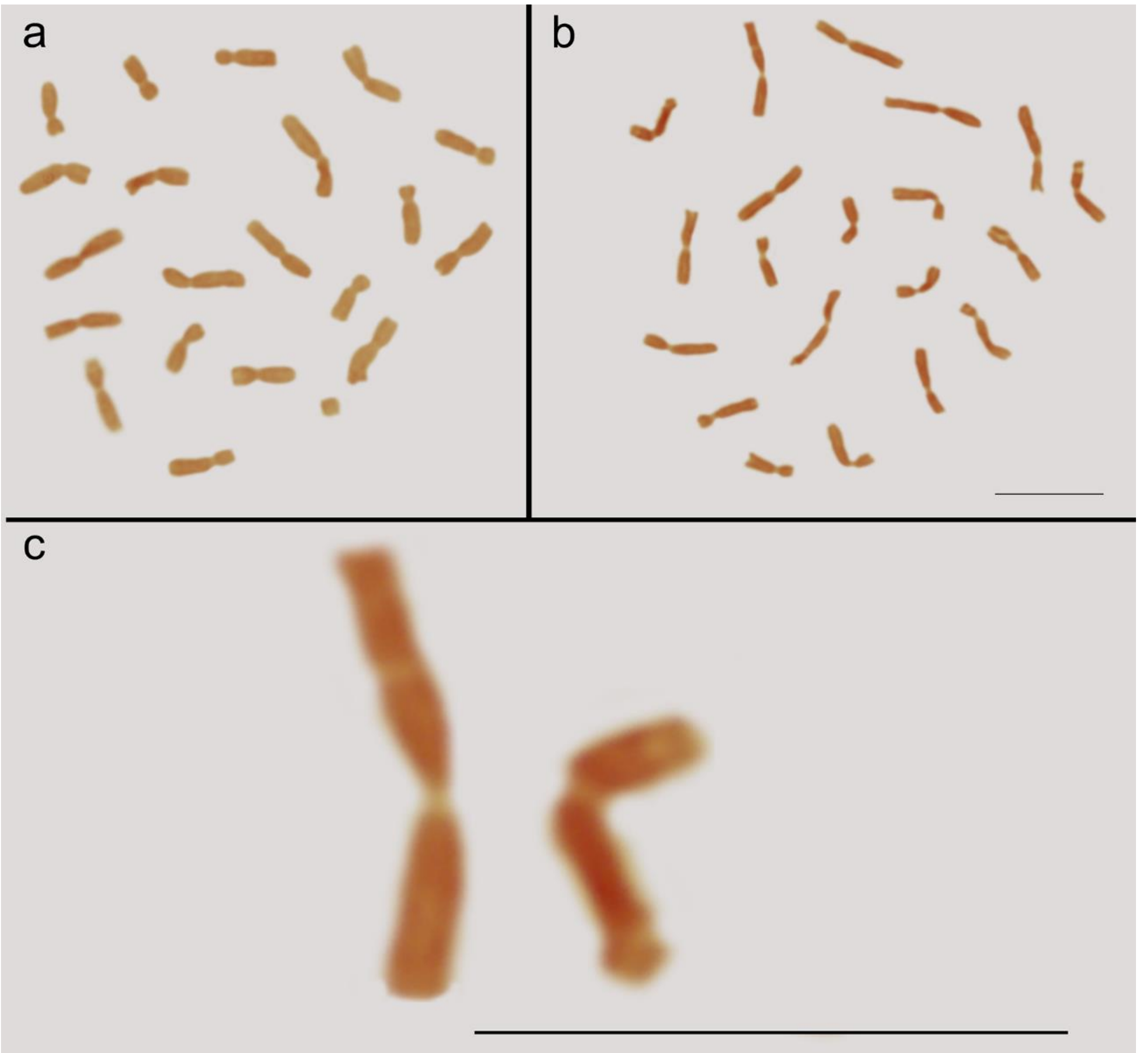

Supplementary Figure 3 – Metaphases of '15-1149-1' stained by the Feulgen reaction. Metaphase evincing **(a)** a chromosome fragment resulted from a deletion and **(b)** translocations. **(c)** Enlarged view of chromosomes that exhibited structural alterations in **b**. Bar = 10 μm. Images were digitized using the Image-Pro Plus software version 6.1 (https://www.mediacy.com/imageproplus).
